# Efficiency comparison of B6(Cg)-Tyr^c−2j^ /J and C57BL/6NTac embryos as hosts for the generation of knockout mice

**DOI:** 10.1007/s11248-021-00248-9

**Published:** 2021-04-12

**Authors:** Yu’e Ma, Lei He, Lijie Xiang, Jie Zhang, Jing Wang, Wenjing Zhu, Wenni Cao, Yichen Zhu, Man Gao, Fei Zhou, Zhiwei Liu

**Affiliations:** CAM-SU Genomic Resource Center, Soochow University, Suzhou, 215123 Jiang Su China

**Keywords:** Chimeric mice, JM8A3. N1, Knockout mice, Germline transmission

## Abstract

Careful selection of the host embryo is critical to the efficient production of knockout (KO) mice when injecting mouse embryonic stem (mES) cells into blastocysts. B6(Cg)-Tyr^c−2j^/J (B6 albino) and C57BL/6NTac (B6NTac) strains of mice are widely used to produce host blastocysts for such procedures. Here, we tested these two strains to identify an appropriate match for modified agouti C57BL/6N (JM8A3.N1) mES cells. When comparing blastocyst yield, super-ovulated B6NTac mice produced more injectable blastocysts per female than B6 albino mice (8.2 *vs.* 5.4). There was no significant difference in birth rate when injected embryos were transferred to the same pseudopregnant recipient strain. However, the live birth rate was significantly higher for B6NTac blastocysts than B6 albino blastocysts (62.7% *vs.* 50.2%). In addition, the proportion of pups exhibiting high-level and complete chimerism, as identified by coat color, was also significantly higher in the B6NTac strain. There was no obvious difference in the efficiency of germline transmission (GLT) when compared between B6NTac and B6 albino host embryos (61.5% *vs.* 63.3% for mES clones; 64.5% *vs.* 67.9% for genes, respectively), thus suggesting that an equivalent GLT rate could be obtained with only a few blastocyst injections for B6NTac embryos. In conclusion, our data indicate that B6NTac blastocysts are a better choice for the microinjection of JM8A3.N1 mES cells than B6 albino blastocysts.

**Supplementary Information:**

The online version contains supplementary material available at 10.1007/s11248-021-00248-9.

## Introduction

The knockout (KO) mouse model plays a critical role in the new era of functional genomics and is widely used to study the role of specific genes. A global scientific collaborative initiative, the International Knockout Mouse Consortium (IKMC), planned to use C57BL/6N-derived ES cells to generate KO models for every mouse gene. As a result, a repository of mutant mES cells has been established to serve the global research community. Based on a collaboration, our center obtained this repository and is now acting to serve the research community in the Asia-Pacifc region. We therefore hold the responsibility to distribute the resource and also to generate as many more mouse models as possible using the mES cells.

There have been several developments that aim to improve the efficiency of generating a genetically modified model using mES cells. Morula aggregation and 8-cell coculture are both accessible techniques that can be used to produce germline transmittable chimeras from C57BL/6N (B6N) ES cells; these techniques are cost effective and do not require extensive training (Gertsenstein et al. 2010; Guo et al. 2014). The 8-cell embryo injection technique has been demonstrated to produce more chimeras and fully ESC-derived mice (Guo et al. 2014; Tokunaga and Tsunoda 1992; Ramírez et al. 2009). Piezo-Micromanipulator microinjection into 4-to 8-cell embryos has been reported to yield a higher rate of male chimeras (Hu et al. 2013). Selecting an appropriate culture medium has also been shown to improve the survival of embryos and ES cells but also elevates the production of founders with high-level chimerism and potential for germ line transmission (Gertsenstein et al. 2010; Ramírez et al. 2009).

The combination of host embryo strains and B6N ES cell lines is crucial for the generation of germline transmittable chimeric founders. Several combinations have been reported as optimal combinations, including BALB/c embryos and B6N ES cells (Alcantar et al. 2016), C57BL/6J blastocysts and B6N ES cells (Fielder et al. 2012), and C3H × BALB/c blastocysts and B6N ES cells (Pacholczyk et al. 2008). It is generally accepted that the inappropriate combination of host blastocyst strains and B6N ES cell lines may lead to less efficient mouse generation and is also time-consuming.

To achieve efficient germ-line transmission with modified B6N ES cells, a range of novel methods have been implemented in an attempt to identify the perfect/ideal host embryo. Taft et al. (2013) developed a “Perfect Host” in which germ cells were ablated in early development, thus resulting in fully ESC-derived chimeric male offspring. Zevnik et al. (2014) reported that the C57BL/6N Albino/Agouti strain not only had a high superovulation response, but also provided a simplified breeding format as a result of identifiable coat color. Although those novel approaches led to some progress, there were also some drawbacks. These modified strains are not as accessible as B6 mice and the cost of using these strains is relatively high. Furthermore, the superovulation responses for some strains were unknown; optimization may be required to improve the superovulation protocol.

B6 albino embryos are frequently used to produce chimeric mice with C57BL/6 ES cells via conventional blastocyst injection method. B6NTac is widely used to obtain a pure B6N background for germline-transmitting mice and genetically modified mouse models. However, there is no published study that directly compares the commonly used strain B6 albino strain and the most widely used strain, B6NTac. To identify ways of improving the efficiency of KO mouse production, in this study, we compared two B6 inbred host strains in combination with JM8A3.N1 ES cells. The aim of our study was to evaluate and select an optimal combination for the efficient production of mouse models.

## Materials and methods

### Mice, superovulation and embryo harvesting

All animals were maintained at 20–26 °C with a light cycle of 12 h D:12 h L and provided with food and water ad libitum in individually ventilated units (Techniplast) in the specific-pathogen free (SPF) facility at Soochow University. All experiments were carried out according to regulatory guidelines for experimental animals approved by the Institutional Animal Care and Use Committee (IACUC) of CAM-SU Genomic Resource Center.

B6NTac and B6 albino females (4–5 weeks-of-age) were injected intraperitoneally with 5 IU/0.1 ml pregnant mare serum gonadotropin (PMSG, NingBO Sansheng Biological Technology Co., Ltd). Then 46–48 h later, these mice were injected with 5 IU/0.1 ml human chorionic gonadotropin (hCG, NingBO Sansheng Biological Technology Co., Ltd). Females were subsequently mated with the same fertile male strains. Embryos were collected at E2.5 by flushing oviduct with M2 medium (Sigma, Cat # M7167) and cultured in M16 medium (Sigma, Cat # M7292) under mineral oil (Sigma-Aldrich, Cat # 8410) at 37 °C under 5% CO2.

### Culture of mES cells

Genetically modified B6N ES cell lines were generated by IKMC and available at the CAM-SU Genomic Resource Center, Soochow University. ES cell clones from the primary frozen archive were thawed and seeded onto feeders in M10 medium (Knockout™ DMEM (Life Technologies, Cat # 10829–018) supplemented with 10% fetal bovine serum, 2 mM L-glutamine (Gibco, Cat # 25030081), 1 × penicillin/streptomycin, 0.1 mM ß-mercaptoethanol and 1000 U/ml leukemia inhibitory factor (Millipore, Cat # ESG1107)) for 2–3 passages in the presence of G418. All cell clones used for microinjection were karyotyped by qPCR using Taqman probes for chromosomes 1, 8, 11 and Y.

### Preparation of mES cells for injection

B6N-derived ES cells were thawed 3 days before injection and cultured in M10 medium in the presence of 2i inhibitors (1 μM PD0325901 and 3 μM CT99021), passaged once when they reached 70–80% confluency and switched back to M15 medium (Knockout™ DMEM (Life Technologies, Cat # 10829-018) supplemented with 18% fetal bovine serum, 2 mM L-glutamine (Gibco, Cat # 25030081), 1 × penicillin/streptomycin, 0.1 mM ß-mercaptoethanol, and 1000 U/ml leukemia inhibitory factor (Millipore, Cat # ESG1107) on the day prior to microinjection. On the day of injection, ES cells were trypsinized and switched back to microinjection media (M15 + HEPES). These cells were kept on ice whilst transferring for microinjection.

### Microinjection and embryo transfer

ES cells were injected into blastocysts using an inverted microscope (Olympus, IX73).

Approximately 10–15 ES cells with small, round and smooth morphology were injected into each blastocyst. After 2–3 h recovery, 7–8 injected blastocysts were transferred into each uterine horn of a 2.5 dpc 8- to 12-week-old psedopregnant ICR female that had been anesthetized by the intraperitoneal injection of Avertin (0.4 mg/g body weight).

### Generation of chimera and GLT breeding

Chimeric mice were identified by coat color at weaning. The combination of JM8A3.N1 ES cells with B6NTac host embryos resulted in agouti/black chimeric mice while the B6 albino host embryos resulted in agouti/white chimeras. At 21 days after birth, the number, sex, and extent of coat color chimerism, was recorded in the offspring. Three male chimeras (6–8 weeks-of-age) with the highest contribution of coat color were chosen as breeders. One male chimera was mated with two B6NTac females. Germ-line transmission in the G1 offspring was confirmed by PCR and subsequent sequencing of the PCR fragment.

### Statistical analysis

Statistical analyses were carried out by using GraphPad Prism version 8.0.2 (Graphpad Software, CA, USA). Data are presented as mean ± SD. Differences were compared by the t-test and *p* < 0.05 was considered to be statistically significant.

## Results

### Embryo yield

The quality of the blastocysts used for microinjection is one of the key factors that influence the generation of KO mice. We collected superovulation data from our center for two years. Next, we compared the yield of embryos after superovulation for the B6 albino with B6NTac strains (Table 1); 8-cell and morulae embryos with normal morphology were counted and then cultured in vitro for further development. The average number of blastocysts suitable for microinjection per female was significantly higher for the B6NTac strain (8.2 ± 4.2) than the B6 albino strain (5.4 ± 2.7) (*P* < 0.0001) (See Table 1).

**Table 1:** Embryo yield arising from superovulation of the B6NTac strain in comparison to the B6 albino strain

	B6NTac	B6 albino	*P* value
No. trials	159	153	
No. Females	900	896	
Average no. 8 cell embryos + morulae	14.0 ± 5.5	11.0 ± 4.5	< 0.0001
Average no. injectable blastocysts	8.2 ± 4.2	5.4 ± 2.7	< 0.0001

### Chimera production

As shown in Table 2, the B6NTac and B6 albino strains resulted in an equivalent proportion of pups (30.2% and 28.4%, respectively; *P* = 0.3890). However, the live birth rate was significantly higher for B6NTac host embryos than for the B6 albino embryos (62.7% *vs.* 50.2%, *P* = 0.0026) (Table 2).

**Table 2:** Comparison of chimera generation by blastocyst injection

	B6NTac	B6 albino	*P* value
No. strains injected	62	56	
No. clones injected	65	60	
No. embryos transferred	1995	3432	
No. recipients	133	230	
Birth rate (pups/embryos transferred)(%)	30.2 ± 12.2	28.4 ± 10.4	0.3890
Live birth rate (live pups/pups born)(%)	62.7 ± 26.0	50.2 ± 19.0	0.0026
Chimeras rate (chimeras/embryos transferred)(%)	13.4 ± 7.9	10.6 ± 5.9	0.0294
> 40% male chimeras rate (> 40% male chimeras/ embryos transferred) (%)	12.4 ± 7.7	9.4 ± 6.1	0.0174
100% male chimeras rate (100% male chimeras/ embryos transferred) (%)	8.0 ± 7.5	1.3 ± 2.5	< 0.0001

**Fig. 1:**
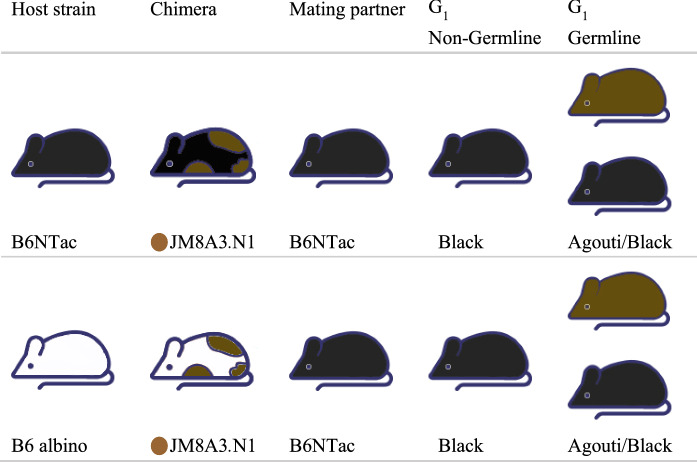
Coat color of chimeras and their offspring (G1) arising from the injection of JM8A3.N1 ES cell into B6NTac hosts and B6 albino hosts

For both combinations, it proved easy to judge the chimerism by coat color (Fig. 1). It was evident that the efficiency of generating chimeric mice varied (Table 2). When analyzed as a percentage of the total number of embryos transferred, the injection of B6N ES cells into B6NTac blastocysts resulted in a significantly higher proportion of chimeric mice (13.4% *vs.* 10.6%, *P* = 0.0294) and a significantly higher percentage of male chimeras with more than 40% agouti coat color (12.4% *vs.* 9.4%). In addition, there was a significantly higher percentage of full colored mice (100% agouti mice) in the B6NTac blastocysts than B6 albino blastocysts (8.0% and 1.3%; *P* < 0.0001). Further study the percentage of chimera formation, when analyzed as a percentage of the number of live male chimeras, the distribution of coat color contribution for the B6 albino embryos ranged from 40 to 99%; this compared to 80–100% in B6NTac embryos (Table 3).

**Table 3:** Comparison of male chimera generation based on the degree of coat colour chimerism

	0–39% (%)	40–79% (%)	80–99% (%)	100% (%)
B6NTac	0.3 ± 2.5	6.4 ± 16.0	32.6 ± 36.9	60.7 ± 37.5
B6 albino	8.7 ± 18.3	39.1 ± 27.8	42.4 ± 28.5	9.8 ± 17.7
*P* value	0.0004	< 0.0001	0.0965	< 0.0001

### Breeding Chimeras for GLT

A total of 125 gene-targeted ES cell clones (65 for the B6NTac host and 60 for the B6 albino host) derived from 118 genes (62, B6NTac; 56, B6 albino, respectively.) were tested for GLT (Table 4). Both host strains resulted in an equivalent percentage of GLT. For B6NTac hosts, chimeric GLT occurred in 61.5% of the clones tested and 64.5% of the targeted genes; these were similar to the data derived from the B6 albino (63.3% and 67.9%, respectively).

**Table 4:** Germline- transmitting chimeras produced from JM8A3.N1 ES cell

	B6NTac	B6 albino
No. Clones test mated	65	60
GLT rate for clones (%)	40/65 (61.5%)	38/60 (63.3%)
No. Strains test mated	62	56
GLT rate for strains (%)	40/62 (64.5%)	38/56 (67.9%)

## Discussion

KO mice are usually generated by the injection of genetically manipulated ES cells into blastocysts. Over recent years, there has been a significant increase in the use of B6N-derived ES cells to produce KO mice. Differing combinations of host embryos and ES cells leads to variation in the ability to produce KO mice (Alcantar et al. 2016; Keskintepe et al. 2007; Pettitt et al. 2009; Schuster-Gossler et al. 2001; Ware et al. 2003), however, the mechanism underlying these findings remains unclear at present. To develop an efficient and easily applicable method to produce KO mice, we compared the efficiency of two widely applied hosts (B6 albino and B6NTac) with regards to the microinjection of B6N (JM8A3.N1) ES cells to generate KO mice.

In order to provide sufficient number of embryos for microinjection, the first step in generating a KO mouse is superovulation; the quantity and quality of the superovulated embryos is critical. It is widely accepted that host strains have diverse responses to hormone stimulation during superovulation. In our experiments, the B6NTac host strain produced more injectable blastocysts per female after superovulation than the B6 albino strain; this finding was consistent with a previous report that described a poor superovulation response in B6 albinos (Schuster-Gossler et al. 2001). Furthermore, the number of injectable B6 albino embryos per female was higher than the number (3.6 per fermale) reported by Alcantar et al. (2016). This major difference may be related to the host age and the embryo collection method. Instead of using mice that were 3–4 weeks-of-age, we used mice that were 4–5 weeks-of-age for ovulation. This was we found in our preliminary research that younger mice produced a sufficient number of embryos but also found that these embryos were poor quality (data not shown). Previously, blastocysts were generally harvested at 3.5dpc; however, we collected embryos at 2.5dpc and cultured these overnight in vitro for further development. We found that this practice achieved better results.

We found that the B6NTac and B6 albino strains produced an equivalent number of pups, but the live birth rate was significantly higher for B6NTac hosts than for B6 albino hosts. Although B6NTac and B6 albino are inbred B6 sub-strains, genetic differences are still known to exist. Zurita et al. (2011) genotyped 12 single-nucleotide polymorphism (SNPs) to distinguish B6 sub-strains and found that 10 of the 12 SNPs were discordant when compared between B6 albino and B6NTac strains. Similar results were reported by Pettitt et al. (2009) in that the B6NTac differed from B6 albino at 16 of the 19 SNPs tested. These genetic polymorphisms may lead to differences in the reproductive performance and superovulation capability when compared between these B6 sub-strains. In addition, poor fecundity of B6 albino hosts was also reported (Alcantar et al. 2016; Pettitt et al. 2009).

Since the first chimeric mouse was produced by the aggregation of two 8-cell embryos, it has been evident that coat color remains the simplest method with which to identify chimerism (Tarkowski 1961). To facilitate coat color identification, the JM8A3.N1 ES cells were derived from B6NTac ES cells by restoring the Agouti locus in one allele (Pettitt et al. 2009). When JM8A3.N1 mES cells are used, chimera founder can be easily identified, regardless of the background of the host strain (B6 albino or B6NTac). It’s well known that the colour of each individual hair is defined by the genotype of the follicle cell and neighbouring melanocyte, thus the chimerism in B6 albino host could be interfered. Regardless of this interference, our data suggest that B6NTac hosts were highly efficient in producing large numbers of chimeras with a high proportion of partial or full agouti coat color as compared to B6 albino. The observed differences in the extent of coat color contribution may indicate that JM8A3.N1 ES cells participation in embryo development is in fact superior in the B6NTac as compared to B6 albino. This observation was consistent with those reported in a previous study (Alcantar et al. 2016) in which the authors demonstrated low efficient in generating chimeras and a high percentage of chimeras using the B6 albino host strain.

However, a high rate of chimerism rate does not necessarily guarantee a higher GLT rate; therefore, we next analyzed the GLT results. A direct comparison between B6 albino and B6NTac has not been conducted in the present study, we did not test all chimeric male mice for GLT; this was consistent with previous reports (Gertsenstein et al. 2010; Pettitt et al. 2009; Cotton et al. 2015; Ware et al. 2003). Generally, three male chimeras with the highest contribution of coat color were chosen to breed (lower level available chimeras were not bred until these failed). If obtained less than 3 male chimeras, all the male chimeras with more than 40% agouti coat color were bred. The GLT data was collected only from the first two litters. It was generally accepted that transmission was detected in either the first or second litter in the vast majority of cases (Cotton et al. 2015). In the present study, both host strains resulted in an equivalent percentage of GLT. Our GLT results are consistent with, if not even more impressive, than that generated by others with other B6N mES cell lines, as well as the rates reported recently for JM8A3.N1 mES cell using other host embryos. Taft et al. (2013) observed an average rate of GLT of 55% for clones when using IKMC B6N-derived ES cells in combination with B6 albino hosts. In another study, Fielder et al. (2012) reported a lower ratio of GLT-chimeric males (36%) when B6N ES cells were injected into B6NTac host blastocysts. Cotton et al. (2015) reported that the GLT rate for JM8A3.N1 clones was 52% using BALB/cJ strain as a host. Based on these data, it appears that B6NTac hosts can achieve similar levels of efficiency of transmitting to germline with only a few injections.

In summary, the two sub-strains of B6 were similar in terms of their ability to generate germline transmittable chimeras for microinjection with JM8A3.N1 mES cell. Nonetheless, the higher live birth rate and better rate of chimerism, render B6NTac a better host embryo strain when used with B6N ES cells. Moreover, the injectable blastocyst yield, birth rate, and GLT data, summarized herein also provide good reference guidelines for other facilities.

## Supplementary Information

Below is the link to the electronic supplementary material.

Supplementary file1 (XLSX 61 kb)

## Abbreviations

KO: Knockout
mES: Mouse embryonic stem
B6 albino: B6(Cg)-Tyr^c^^−^^2j^/J
B6NTac: C57BL/6NTac
B6N: C57BL/6N
GLT: Germline transmission
